# Uncertainty and objectivity in clinical decision making: a clinical case in emergency medicine

**DOI:** 10.1007/s11019-016-9714-5

**Published:** 2016-06-03

**Authors:** Eivind Engebretsen, Kristin Heggen, Sietse Wieringa, Trisha Greenhalgh

**Affiliations:** 1 Institute of Health and Society, Faculty of Medicine, University of Oslo, Box 1030, Blindern, 0318 Oslo, Norway; 2 Nuffield Department of Primary Care Health Sciences, University of Oxford, Radcliffe Primary Care Building, Radcliffe Observatory Quarter, Woodstock Road, Oxford, OX2 6GG UK

**Keywords:** Evidence-based medicine, Uncertainty, Clinical reasoning, Standardization

## Abstract

The evidence-based practice and evidence-based medicine (EBM) movements have promoted standardization through guideline development methodologies based on systematic reviews and meta-analyses of best available research. EBM has challenged clinicians to question their reliance on practical reasoning and clinical judgement. In this paper, we argue that the protagonists of EBM position their mission as reducing uncertainty through the use of standardized methods for knowledge evaluation and use. With this drive towards uniformity, standardization and control comes a suspicion towards intuition, creativity and uncertainty as integral parts of medical practice. We question the appropriateness of attempts to standardize professional practice through a discussion of the importance of uncertainty. Greenhalgh’s taxonomy of uncertainty is used to inform an analysis of the clinical reasoning occurring in a potentially life threatening emergency situation with a young patient. The case analysis is further developed by the use of the Canadian philosopher Bernard Lonergan’s theory about understanding and objective knowing. According to Lonergan it is not by getting rid of or even by reducing uncertainty, but by attending systematically to it and by relating to it in a self-conscious way, that objective knowledge can be obtained. The paper concludes that uncertainty is not a regrettable and unavoidable aspect of decision making but a productive component of clinical reasoning.

## Introduction

More than 20 years ago while one of the authors (KH) was working as a clinician on an acute hospital ward, she had an experience she has since reflected on again and again. Recently, she began to relate the experience to uncertainty. The case is summarized below, but in order to protect the patient and family’s confidentiality, certain characteristics have been omitted and others changed.

Kristin had recently passed the national certification test for nurse anesthetist and was employed as a trauma team member. One evening, the team received a call from a father who appeared to be terrified, reporting that his young adult son was lying unconscious on the floor. As Kristin was running towards the ambulance, the doctor on call asked Kristin to be prepared for a cerebral hemorrhage. To experience a young person with acute brain injury was what Kristin (a newly qualified specialist clinician) feared the most. She tried to remind herself about the standard protocol, and wondered if she was up to date on recent protocol changes. This made her nervous. The situation was stressful; she knew she had to be calm and focused both to cope with the situation and convey to the patient and family the impression of being a skilled professional. She repeated for herself the symptoms characteristic for cerebral disorder and what she hoped was the hospital’s current guidelines for immediate treatment. And she knew all the questions the team would expect her to report on as well as giving them precise information about the emergency scene and the patient’s condition. By the time she approached the front door of the house, which was being held open by the father, Kristin felt quite confident.

As she ran up the few steps to the entrance, something strange happened. Kristin caught a glimpse of a woman (probably the young man’s mother) sitting at the kitchen table with a cup of coffee. This remarkably calm behavior of a family member contrasted with the terrified impression given by the young man’s father on the phone. Kristin still has a vivid memory of how this astonishing impression heightened her awareness to signs suggesting that the unconscious young man might *not* be suffering from a brain hemorrhage. She found the young man lying on the floor in a poorly lit room. His respiratory rate was normal, as was his blood pressure and pulse. He did not vomit, but responded feebly when Kristin tried to make verbal contact with him. He was cold and had perhaps been lying on the floor for a while, which was also somewhat strange, given that his parents were at home. She asked the young man’s father whether he knew about any illness history and use of medication. The father murmured some unclear words, which Kristin interpreted as a sort of unwillingness to answer. She looked around in the room for a couple of seconds without finding evidence of pills and not knowing what else to look for or question about. As she left the house ready to bring the patient to the hospital, she asked if the father would go with the ambulance to the hospital. No clear answer. The ambulance left with the blue emergency lighting and without the father.

To cut a long story short, the young man had taken an overdose of medication in an apparent attempt to commit suicide.

This experience still occupies Kristin’s mind and inspires her interest in clinical reasoning, the intuitive side of expertise, and the role of uncertainty in clinical decision-making. There are several interesting aspects of her uncertainty in the above case. One is the notion she originally held—that being a professional gives little or no space for uncertainty. Rather, being professional entails (we often assume) being fully abreast of the situation and ready for action. Decisive action might of course be sensible in a life threatening situation, but even when Kristin anticipates the report she will have to provide to the trauma team, she is initially convinced that only clear and exact answers will be expected and there will be no place for vagueness or guesswork. Indeed, uncertainty in clinical practice is increasingly viewed as something to be resolved rationally, given the growing emphasis on evidence-based medicine (EBM) and standardization of health care.

Greenhalgh (2013) proposed a conceptual taxonomy of uncertainty—uncertainty about the evidence (e.g. what do the guidelines show?), about the narrative (what is the patient’s story?), about case-based reasoning (what best to do in the circumstances?) and about multi-professional working (how best to communicate and collaborate?)—that resonates with several aspects of Kristin’s experience. When she does not fully recollect the relevant clinical protocol, she experiences uncertainty in the evidence *“*where key questions relate to the completeness, accuracy, and relevance of research-based evidence*”* (Greenhalgh 2013: 41). Secondly, Kristin also identified narrative uncertainty, relating to the patient’s story, which lacked coherence (for example, the mother’s calm demeanour, the father’s incoherent response to Kristin’s questions and his seeming unwillingness to go with the ambulance to the hospital). Kristin’s hesitation about what sort of signs to look for and how she best can inform her clinical judgement is an example of *what best to do in a particular situation with this patient*. The fourth category, uncertainty about how a multidisciplinary team might co-ordinate complex care, is illustrated by Kristin’s thoughts about how the team will respond, knowing what she believes to be their expectation for precise and clinically focused answers. Would it be appropriate to reveal information of uncertain relevance—for example about observed circumstances in the house? Would Kristin’s observations of the coffee drinking mother and the father’s unwillingness to answer contribute to a wider assessment of this case by the clinical team? Initially, Kristin thought no.

Kristin could have searched to manage her uncertainties and standardize her decisions through the use of formal probabilistic assessments and Bayesian reasoning (Medow and Lucey 2011). She could for instance have attempted to estimate the probability of the patient having cerebral hemorrhage given specific signs, symptoms or test outcomes based on information about prevalence or frequency of the condition within the relevant population and information about the accuracy of the test. Whilst such an approach might have reduced the uncertainty around some aspects of the evidence (Greenhalgh’s first type) it would not address all the other aspects of uncertainty present in Kristin’s situation. More importantly, the Bayesian approach builds on the assumption that controlling uncertainties (through probability estimates) is the best way of dealing with them. In this paper, we present a different perspective on uncertainty: by exploring the different types of uncertainty present in Kristin’s case, we raise the question whether any of these uncertainties might have played a productive role in her decision making. This means shifting focus away from the dominant view of uncertainty as a threat to evidence-based decisions into viewing uncertainties as generative to informed decision making [as Locke et al. (2008) have done in relation to making doubt generative in the research process]. More specifically, we will unpack the case presented above drawing on Bernard Lonergan’s theories about understanding and objective knowing and thereby explore possible subversive consequences of an increased standardization of health care.

## Standardization and ideals of evidence-based medicine

During the last 50 years there has been a strong movement towards standardization of medical practice through protocols and clinical guidelines. Different methodologies have been used to develop such documents with the common ambition of creating predictability, accountability and objectivity by streamlining processes (Timmermans and Berg 2003). In the 70s and 80s, the dominant way of reaching agreement about how to manage clinical situations was the consensus panel, made up of experts who produced ‘consensus statements’ based on a form of collective reasoning that was generally opaque and unauditable. But from the early 1990s, the evidence-based practice (EBP) and evidence-based medicine (EBM) movements worked to produce new, standardized and auditable guideline development methodologies based on systematic reviews and meta-analysis of best available research. According to Sackett’s much cited definition, EBM is “the conscientious, explicit and judicious use of current best evidence in making decisions about the care of individual patients” (Sackett et al. 1996). In line with this program statement, EBM has emphasized the use of clinical guidelines and challenged clinicians to question their reliance on pathophysiological reasoning and unenhanced clinical judgement. Clinicians should instead be trained in reading research literature and converting the findings from published studies into probabilities according to Bayesian principles of reasoning (Solomon 2015). Behind EBM/P’s search for standards and methods is an ambition to “meet a need for ‘certainty’ and ‘structure’ in many professionals and consumers alike” (Cluett 2006: 52).

Arguably, the overall aim of EBM is to reduce uncertainty through the use of standardized methods for knowledge evaluation and use. With this drive towards uniformity, standardization and control comes a suspicion towards intuition, creativity and uncertainty as integral parts of medical practice (Greenhalgh 2013, 2014). This paper questions the productivity of the attempts to standardize professional health care through a discussion of the importance of uncertainty. We claim that uncertainty is not a regrettable and unavoidable aspect of clinical decision making but a productive component. We will build on the Canadian philosopher Bernard Lonergan as well as analysis of the emergency case and demonstrate the importance of uncertainty for effective decisions.

## Bernard Lonergan’s hermeneutics of knowing

Bernard Lonergan (1904–1984) was a Canadian theologian, philosopher, mathematician and economist. Although he has perhaps had most impact within theological circles, he has also made major contributions to the hermeneutics of science with significance far beyond the domain of theology. In his principal work *Insight. A study of human understanding* [1992 (1957)] he developed a general theory of how knowledge comes about. His ambitious aim was to create a general method of understanding, a so called generalized empirical method (GEM), applicable within all science and practical reasoning. Lonergan’s approach should be distinguished from the positivist thesis of the unity of science, which claims that common scientific laws apply everywhere and might be explored through methods drawn from natural science. Lonergan’s claim is rather the opposite: all science involves interpretation and therefore needs methods not only for measurement and explanation but for how to perform interpretation as part of the scientific endeavor. Here, science might draw on insights from hermeneutics and the human sciences. This need for interpretation does not only concern science in a strict sense but also clinical practice (Engebretsen et al. 2015).

Lonergan’s work is a criticism of what he considers to be the domination of empiricism within modern science, a criticism that resonates with the characteristic of EBM as “the last bastion of crude empiricism” (Greenhalgh 2013: 5). He refutes the idea that there is “an already out there now real” that science should try to mirror (Lonergan 1992: 276). However, Lonergan’s argument is not merely a social constructionist claim that our knowledge is historically and culturally situated and thus in its most extreme consequence, never objective. Lonergan is a firm believer in objective knowledge. But to Lonergan, objective knowledge is not characterized by being in correspondence with the world out there. Lonergan is an idealist, not an empiricist. He sees objective knowledge as rooted in our mind, not in experience. His “primary concern is not the known but knowing” (Lonergan 1992: 12). Objective knowledge is an endeavor, a verb not a noun. It is enacted through the process of inquiry, not discovered. Lonergan understands knowledge as performative, but in strictly idealist and not social constructionist sense. Unlike more recent scholars within the science and technology studies tradition (e.g. Law and Mol 2002), Lonergan is not primarily concerned with how scientific reasoning is constituted through practice but considers scientific reasoning as a practice in itself. Reasoning is a technology, a craft. And it is the quality of the reasoning that determines the quality of the knowledge. In the above case example, Kristin does not *discover* a set of pre-existing facts about the patient’s situation. Rather, she *builds knowledge* by working with the clues as they emerge and putting them together. And once this knowledge exists in Kristen’s mind, it can no longer be reduced to the clues that started off the process searching for insight. The knowledge does not correspond with “what really happened”. Through the performance of knowing, the “what really happened” has changed in an important way: it has become infused with meaning. This meaning is not part of the events themselves but added by Kristin through her act of inquiry. Kristin creates links between events such as the patient’s symptoms, the mother sitting at the kitchen table, the father’s unwillingness to answer, and the young man’s cold body indicating that he has been lying alone in his room for a while. Furthermore, she organizes the events into categories based on what she has experienced before and the observations broaden her awareness and activate a doubt about the standard protocol she was depending on as she entered the house. The “meaningful whole” that Kristin makes from the situation is certainly based on real events, but it is not *equal to* those events. There are events which are left out from the picture she draws and there is information added to the events in terms of knowledge about similar situations, relevant medical conditions, different sets of guidelines and procedures etc. In short, Kristin’s knowledge is the result of a complex intellectual endeavor, an act of creativity as much as an act of observation.

Moreover, the knowledge she produces is (in a sense) instant. It is created in the situation; it is a unique act of knowing. Kristin does not address the complex clinical problem through the simple application of evidence. Rather, her knowledge is born out of her appraisal of an extraordinary event or situation, which opens her eyes for new signs which trigger her intelligence. It is the result of an insight. Lonergan explains the notion of insight with a detective story:

In the ideal detective story, the reader is given all the clues yet fails to spot the criminal. He may advert to each clue as it arises and needs no further clues to solve the mystery. Yet he can remain in the dark for the simple reason that reaching the solution is not the mere apprehension of any clue, not the mere memory of all, but a quite distinct activity of organizing intelligence that places the full set of clues in a unique explanatory perspective.

By insight, then, is meant not any act of attention or advertence or memory but the supervening act of understanding (Lonergan 1992: 3)

An insight cannot be forced. It can be prepared for through the collection of clues. But the insight itself is not an action but a cognitional event; it is the moment when the clues fall into place. According to Lonergan, it “comes suddenly and unexpectedly” and it is “a function not of outer circumstances but of inner conditions” (Lonergan 1992: 28). Objective knowledge is thus not merely conditioned by the world out there but a cognitional event. This way, it is by definition uncontrollable. Objective knowledge has the character of event and surprise and is thus in a fundamental sense based on prior uncertainty. This point bears close resemblance with Gadamer’s theory of “understanding as event” (Gadamer 2004). However, through his concept of insight Lonergan puts more emphasis on the self-conscious character of this event than Gadamer does. Though sudden and unexpected, insights are *never* unnoticed. An insight presupposes meta-knowledge and meta-cognition. Lonergan understands knowledge as “the personal appropriation of one’s own rational self-consciousness” (Lonergan 1992: 769). We obtain objective knowledge by attending to how our mind operates when knowledge is obtained. Objectivity is self-appropriation. It is the result of a gaze turned inwards not outwards. Thus it does not exclude uncertainty but encompasses it. It is not by rationally resolving or even reducing uncertainty but by attending systematically to it and by relating to it in a self-conscious way, that insightful objective knowledge can be obtained. Lonergan’s idea of method implies the self-appropriation of uncertainty rather than the reduction of it.

Lonergan’s method implies attending self-consciously to our own insights on three different levels which he calls experiencing, understanding and judging. Hence, the act of knowing is not one act but a set of cognitional operations. It refers to a cognitional apparatus. As we shall see, uncertainty is an integral and productive part of all these different activities.

## Experiencing, understanding and judging

According to Lonergan, all knowing starts with a set of data. The dataset is the subject matter of our understanding; it is what triggers our understanding but is not yet understood; what calls for explanation but is prior to any explanation. Hence, the experiencing phase takes place before the real knowing process starts. Yet, to delimit the experiential basis for our understanding is not an easy task and a major challenge in EBM. Answering the question “what counts as data?” is not merely about passive recording of sensations but also an active process of imagination. By imagination, we mean an ability *to see in your mind’s eye* what is in the forefront of a situation, what combination of various forms of knowledge (usually several) is of relevance for acting here and now as well as arise awareness for possible future consequences (Sutphen and Heggen 2015). In classical rhetoric, this faculty was called “evidentia” referring to the ability to produce a visual impression on the listener through the “eyes of the mind” (Vasaly 1993). Thus experiencing is not just about recording and sorting out perceptions, but it is also about picturing information that is not immediately present to the senses.

When Kristin arrives at the emergency scene, she must observe: a young man unconscious on the floor; a woman sitting at the kitchen table drinking coffee; the father hardly answering when he is asked about his son. She must also transcend her immediate sensations through the use of her imagination: medication, despair, cold body, unwillingness to answer questions—all these are factors that are possibly present on the scene but not immediately observable.

Still, imagination is pre-reflective. It does not involve meta-knowledge in terms of self-awareness about own knowing and is thus prior to real understanding. As a clinician, you have to learn and trust your ability to enter a clinical scene with an open mind but still in a state of readiness to respond. An open mind is different from an empty mind and different from asking questions. It is a state of free floating awareness.

However, already on this level, there is a form of uncertainty involved: experiencing is about more than simple recording of data that are present to the senses. The ability of adding information that is not seen but imagined introduces an element of uncontrollability and uncertainty into the process: *there is more to the case than you can see*. Without creativity and imagination, the clinician risks overlooking important information.

When the clinician starts making sense of her data and thus enters the understanding phase, a new element of uncertainty is introduced, *the question*. Our intelligence unfolds as an internal conversation, through asking and answering questions (Lonergan 1992: 33–34). By asking questions we become aware of what we do not know and what we want to know; we are naming the unknown. Hence, the question represents the appropriation of our uncertainty. The act of questioning draws our attention to what we do not know. At the same time, the question orients us towards an answer. By asking questions we turn our images into clues, like the detective in Lonergan’s example. While knowing what we do not know, we simultaneously know that knowledge can be obtained. Hence, the question mediates between the known and the unknown by identifying the “known unknown” (Lonergan 1992: 555). By asking a question we anticipate an answer. Thus, questions are necessarily based on presuppositions or heuristics. According to Lonergan, heuristics are anticipations of the known while still unknown, they are horizons of expectation that guide our inquiry for knowledge (Lonergan 1992: 60–62). This differs from searching for guidelines or standards for best practice. We can only reach an answer by anticipating and believing what is not certain. In sum, our intelligence can only prepare itself for new insights by maximizing our uncertainty through questions. Furthermore, questions can be posed only by building on uncertainty in terms of beliefs and anticipations. Uncertainty is thus the vehicle in our inquiry for new knowledge.

In our case, Kristin’s inquiry for knowledge starts even before she enters the emergency scene. In the moment she receives the call and thus records the first pieces of data, she begins her questioning: *what might this be*? The first piece of data given to her apart from the basic initial details is the interpretation of a colleague in the trauma team: be prepared for a cerebral hemorrhage. Kristin starts questioning this information based on heuristics, i.e. already acquired knowledge about similar conditions. This way she is starting the process of defining what she does not know, while at the same time directing her mind towards a possible answer. Her presuppositions guide her questioning: Headache? For how long and with what characteristics? Vomiting? Unconsciousness—for how long? Any known history of illness? And so on.

Through this process, sensations and images are transformed into ideas, concepts and definitions. While images are the results of imagination, ideas are products of intelligence, according to Lonergan (1992: 32). Ideas are self-conscious images. Concepts are, in their turn, ideas that are formulated explicitly and expressed in words and symbols. While images are immediate and automatic, ideas imply work, efforts, and conscious activities. At the same time, ideas also presuppose an aspect of luck or gift. Ideas come about through insights. You cannot force them to come; you can only create the conditions where the insights are likely to occur. There is always an element of surprise and guesswork involved.

The mind work involved in understanding is clearly reflected in our case: Kristin works on the data by repeating for herself the symptoms characteristic for cerebral disorder, by reminding herself about the standard protocol and what sort of information she should bring to the trauma team. At the same time, the insight does not occur on command but in a “glance”. And what is particularly interesting with Kristin’s insight is that when it occurs, it changes her whole line of questioning. In a glance, she understands that the direction of her questions has been mistaken. Lonergan has named this particular kind of insight “inverse insights” (Lonergan 1992: 43–50). This type of insight is characterized by the realization that we have been asking the wrong questions, we have been barking up the wrong tree. The anticipations on which we build our questions are mistaken. With Kristin’s reverse insight a new set of questions is launched (Maybe it is a suicide attempt or deliberate self-harm?) and with these questions, old data gain new meaning (the young man being unconscious due to drug related overdose) and new images call for interpretation (the father’s unwillingness to give information about his son’s condition for instance). Without re-orienting her questions, Kristin would not have been able to get the clue. This shows that adequate interpretation involves active awareness about the possibility of misinterpretation. Derrida has latterly echoed this argument in his theory about the possibility of misunderstanding as a condition for all understanding (Derrida 1997). The knower needs to be aware that any piece might also fit into a different or bigger puzzle. The answers she gets are products of *her* line of questioning and a different set of questions might produce different answers. This is not the same as claiming that all answers are equally good. Rather, it is a reminder that the knower must look critically at his questions, not only his answers. A true insight should take into account that not only might its result be wrong but it might also be sought in the wrong direction, by asking inadequate questions. An insight is thus based on the possibility of its own inversion. In more practical words, the mind must be prepared for the possibility of inverse insights—if not, they are unlikely to occur.

Lonergan also refers to this process of questioning the data as abstraction, and he uses this term in a different way from usual. Based on the argument above, Lonergan claims that abstraction is not only about distilling the essence of the data by putting aside aspects that are considered irrelevant. Abstraction also involves adding on to the data, or enriching them by organizing them into already established patterns or puzzles of knowledge (Lonergan 1992: 111–112). Thus, understanding does not occur through a process of purification where the data are washed clean of any outer interference. Rather, the meaning of the data is brought into being through our intellectual processing and creative nourishing of the data. Hence, although Kristin’s first preliminary assumption (that the diagnosis was cerebral hemorrhage) was incorrect, she followed the right procedure by drawing on her presuppositions to frame the data. The key to her success was that she acknowledged the uncertainty of her initial conclusions and the reversibility of her insights.

Lonergan differs from other epistemologists by distinguishing between an understanding mode and a critical mode in the inquiry for knowledge, between understanding and judging. Understanding captures possibilities; we are performing a kind of brainstorming by expanding our vision and testing out drafts of interpretation. However, in order to fulfill our inquiry for knowledge, we must also appropriate the understanding and commit ourselves to a particular interpretation. The knower must explore possibilities, but he must also take a stand; he must evaluate and verify the knowledge. Descartes did not differentiate between these different modes of knowing which made him conclude that the clearness and unambivalence of insights were criteria for their truthfulness. According to Lonergan, questions seeking for clearer understanding are analytically distinct from questions asking for the trustworthiness of the understanding (Lonergan 1992: 106). By mixing them up, like Descartes did, we risk both limiting the creativity of the understanding process and the criticism of the judging process.

Arriving at the emergency scene, Kristin does not ask questions only in order to expand her understanding, she also asks questions of reflection in order to verify her interpretation: Is her new interpretation correct? Is it a suicide attempt? These different sets of questions anticipate different answers. While her questions of understanding are exploring new information, like illness history, medication, pulse and blood pressure etc., her questions of reflection are looking for verification: It is a suicide attempt or it isn’t. Is it eventually accidental or intentional? Her answer is probable, possible, likely or undeterminable. Lonergan emphasizes the argumentative aspect of this operation: it is an *act of weighing* (Lonergan 1992: 304)*. The judgment is not given from the facts. B*y making a judgement, the doctor commits himself to one out of several possible interpretations. The judgement makes the inquirer self-accountable, according to Lonergan. It implies taking the responsibility for a specific interpretation and at the same time admitting that the interpretation could have been different. It is an acknowledgement of uncertainty.

However, as earlier stated, Lonergan does not reject the notion of objectivity. Rather, he redefines it. Objectivity is an intellectual phenomenon, according to Lonergan. It is obtained by reflecting back on the operations that you have done in order to obtain understanding asking the reflecting question: “Did I get it right?” You cannot validate your knowledge by measuring the correspondence between your knowledge and the “world out there”, but by inspecting the methods through which you formed your knowledge. As soon as Kristin has formed her knowledge, she cannot return to the data “as they were” before the process of inquiry started. To measure the interpretation against some pure and untouched data prior to interpretation, is thus an impossible task. What she can do, however, is to look back on her operations of understanding. Lonergan differs between three concepts of objectivity which are interdependent. *Empirical objectivity* or correct experiencing is the first form. Here the essential judgment is: “did I observe and/or imagine the situation in the right way?” This form of objectivity is however incomplete in itself and must be complemented by *normative objectivity* which depends on the following judgement: “have I asked every conceivable question?” Still, this judgement must be followed by a last judgement, which ensures *absolute objectivity*: “Have I asked the right questions?”(Lonergan 1992: 402–407).

This way, it is not the result which is or is not objective but the process. Objectivity is the name of the judicious and transparent awareness of all operations involved in the process of knowing. It is by reflecting back on this process (Are my observations right? Have I asked all the questions? Have I asked the right questions?) that objectivity is ensured.

This process does not necessarily result in a clear cut conclusion which rules out all uncertainty and doubt. Rather is implies certainty about any possible uncertainties; I am sure that I have taken all aspects into account and that this is as far as I can get in the process of inquiry. Absolute objectivity is not absolute certainty. Rather is it a judgement which is absolute in the sense that it takes all aspects—certainties as well as uncertainties—into account.

## Uncertainty and decision making

According to Timmermanns and Berg, “standardization has penetrated every corner of medicine” (2003: 3). EBM aims to reduce uncertainty through the use of standardized methods for the evaluation and use of knowledge. The aim of this paper is to argue the opposite, namely the importance of uncertainty for making objective clinical decisions. We will especially emphasize the following four aspects of uncertainty:

### Imagination

Following Lonergan, the experiencing phase starts before the real knowing process and is not a passive recording of data as if one is following a standard procedure. It is an active process of imagination, and a vivid awareness about the situation you are a part of. Imagination is an ability to see in your mind’s eye and picturing information that is not immediately present to the senses (as Kristin perceives that there might be something in the relationship between the son and his parents). This ability to act with awareness for additional not seen information implies uncertainty.

This initial phase of understanding is pre-reflective, not involving conscious questioning about your knowing. As a health care provider you have to trust your ability to enter a clinical scene with an open mind which is different from a distant observational clinical gaze, at the same time as you are ready to respond and act.

### Reflective questioning

As Lonergan explains, our intelligence can only prepare itself for new insights by maximizing our uncertainty through questions. When asking questions we become aware of what we do not know and want to know and can direct our attention in order to seek knowledge. The question is in itself a new aspect of uncertainty and a driver for knowledge. By questioning you identify the “known—unknown” as expressed by Lonergan. Kristin works with what she does not know, with active questioning, in order to obtain knowledge about the situation.

### Understanding as an event and surprise

A health care provider has to be prepared for the possibility of inverse insights meaning that both question and answer might be inadequate. Without introducing this aspect of uncertainty inverse insights and surprising answers are not likely to occur. This insight does not occur on command but in a “glance”. And what is particularly interesting with Kristin’s insight is that when it occurs, it changes her whole line of questioning. At that moment, she understands that the direction of her questions has probably been wrong. Hence, understanding involves active awareness about the possibility of misinterpretation.

### Critical judgement

Critical judgement is an introduction of uncertainty as a weighing of argument underpinning your interpretation. In Kristin’s case it is a verification of whether or not the young man is unconscious as a consequence of a suicidal attempt or a cerebral hemorrhage. It is about taking the responsibility for one’s interpretation and at the same time acknowledging that the interpretation could have been different.

EBM does not provide any instrument for handling the complexity of real world situation. It offers only simplification through the use of standards. This search for standardization and minimization of uncertainty risk producing adverse effects: As Lonergan has shown, it is by drawing on our uncertainties that knowledge based decisions are possible. The lack of awareness of the different aspects of uncertainty involved in decision making might therefore hamper an objective clinical decision.

## Uncertainty and objective knowledge

In the EBM literature, the relationship between uncertainty and objective knowledge is often understood as dichotomous. The dominant question is: how can we reduce uncertainty by building on standards and protocols that ensure objective judgement? Drawing on Lonergan, we claim that this question is misleading and based on a mistaken understanding of objective knowledge. Rather than searching for objectivity through standardization, streamlining and reduction of uncertainty, we claim that objective knowledge can only be obtained by attending to one’s uncertainty on different levels of the process of inquiry. EBM is built on an empiristic understanding of objective knowledge as the simple correspondence between perceptions and “the world out there”. Our diagnostics and treatment of the patient shall correspond to the “real” condition. This may be obtained, it is assumed, by following standardized recommendations built on clinical research. As opposed to this view, we claim that objective knowledge can never be obtained by just following the protocol guided by research. Imagination and creativity are prerequisites for objective knowledge. Objectivity can only be obtained through imagination, reflective questioning, openness for surprises and critical judgement. Hence, objectivity is in a certain sense the opposite of protocols and standards. Objectivity is instant knowledge (Lillehagen et al. 2016), it exists *here and now*, it is a unique event occurring in a particular situation and setting. Thus objectivity is *per se* a risky business. Objective knowledge involves uncertainty; it implies taking a risk and being taken by surprise.

What we suggest is thus a different conception of evidence than the one dominant within EBM. We do not refute the concept of evidence; on the contrary, we embrace it and redefine it. Evidence—or objective medical knowledge—is not a package capturing the world independent of the concrete situation and setting. Evidence is an endeavor; it is a concrete act of knowing or “evidence basing” (Bohlin and Sager 2011). Thus it cannot be obtained by simply following an “evidence-based” guideline or protocol. It must be created here and now. Evidence is thus about attending consciously to the process of inquiry and in a sense, similar to a judge attending consciously and transparently to the procedures through which he reaches his conclusion. In legal theory, the right decision is not equal to the law. The right decision does not follow from the sources (the law) but must be taken in a concrete situation following a certain procedure (legal methodology). The right decision is built up through a structured, self-conscious and transparent endeavor, not by simply following the law. We believe that EBM needs a similar attention to *how* evidence comes about in a concrete situation and not only *what* evidence is in terms of the best available sources/standards of knowledge.

## Postscript

This paper has considered clinical uncertainty from the perspective of the philosophy of clinical knowing but there are many parallels in the field of research—and a parallel literature in the philosophy of scientific knowing. Sir Peter Medawar, for example, wrote about the crucial role of imagination and surprise in the generation and testing (respectively) of scientific hypotheses (Medawar 1969). More recently, Locke et al. (2008), writing in the field of organisational case study and drawing on the pragmatist philosopher Charles Pierce, have considered the role of doubt in the research process. Like Lonergan, Locke et al. view doubt not as an undesirable phenomenon that needs to be resolved through validation but as an essential component of the scientific method. Without doubt, and the careful reflection on doubt, the process of conjecture essential to scientific discovery cannot begin. Just as evidence-based medicine sometimes wrongly assumes that clinical reasoning occurs only or primarily by deduction from ‘facts’, so a naïve framing of scientific research wrongly assumes that scientific deduction holds primacy over scientific *abduction* (the act of asking “what is there here to explain?” and “what might explain what needs to be explained?”). But that is a subject for another paper.
